# CRISPR-based platform for carbapenemases and emerging viruses detection using Cas12a (Cpf1) effector nuclease

**DOI:** 10.1080/22221751.2020.1763857

**Published:** 2020-06-02

**Authors:** Lucia Ana Curti, Federico Pereyra-Bonnet, Guillermo Daniel Repizo, Jessica Vannina Fay, Karina Salvatierra, María José Blariza, Daiana Ibañez-Alegre, Adriana Raquel Rinflerch, Marcos Miretti, Carla Alejandra Gimenez

**Affiliations:** a INPA-CONICET- Universidad de Buenos Aires, Argentina; b CASPR Biotech, San Francisco, CA, USA; c Facultad de Ciencias Bioquímicas y Farmacéuticas, Departamento de Microbiología, Instituto de Biología Molecular y Celular de Rosario (IBR, CONICET), Universidad Nacional de Rosario, Argentina; d Laboratorio GIGA, FCEQyN, Instituto de Biología Subtropical, Universidad Nacional de Misiones – CONICET

**Keywords:** Infectious diseases, molecular diagnostic, CRISPR/Cas12a, RNA viruses, antibiotic resistance

## Abstract

CRISPR-Cas12a (also called Cpf1) has been commonly used for genomic editing, based on its ability to generate precise double-stranded DNA (dsDNA) breaks. Recently, it was demonstrated that Cas12a exhibits unspecific ssDNAse activity upon target recognition. This feature allows CRISPR-Cas to be coupled with a ssDNA reporter and generate a fast, accurate and ultrasensitive molecular detection method. Here, we demonstrate that Cas12a was able to detect DNA target sequences corresponding to carbapenemases resistance genes such as KPC, NDM and OXA. Also, with the addition of a reverse-transcription step, we were able to detect viral RNA sequences from DENV, ZIKV and HANTV genomes. In all cases, assay run time was less than two hours. Additionally, we report attomolar levels of detection. This methodology was validated using clinical samples from patients infected with Dengue virus. Reactions were visualized by detection of a fluorescent signal, as well as by the use of a simple lateral flow strip. These results indicate that Cas12a is able to detect both DNA and RNA targets, making it an appropriate and convenient tool to detect all types of pathogens.

## Introduction

Worldwide disease outbreaks have exposed the need for and challenges associated with the development of diagnostic tests, specifically infections caused by viral and multidrug-resistant bacteria (commonly known as superbugs). The incidence of newly emerged or re-emerging infectious diseases, global antimicrobial resistance, and food and environmental contamination continues to increase, especially in underdeveloped countries or resource-limited regions [1]. Therefore, there is an urgent need to develop detection technologies with high sensitivity and specificity that can be used for rapid and versatile point-of-care (POC) diagnostic applications. In this context, antigen-based rapid diagnostic tests require minimal equipment and thus provide important benefits for patients located in geographic areas where sophisticated molecular test are not available. On the contrary, they show limited sensitivity and specificity, and since they depend on the host antibody response they apply better for epidemiological studies [2]. To overcome these drawbacks, nucleic acid-based detection methods such as RT–PCR have been designed. Although they are sensitive and rapidly adaptable, most require extensive sample manipulation and expensive machinery making it impossible to implement in underdeveloped countries [3,4].

CRISPR-Cas effector nucleases have been used for many biotechnological applications [5]. Within the CRISPR-Cas effectors family, Cas12a (previously called Cpf1) is an RNA-guided DNase belonging to the class II Type V-A system [6]. Cas12a mediates robust specific RNA-guided double-stranded DNA (dsDNA) cleavage and it was recently discovered that it triggers an indiscriminate single-stranded DNA (ssDNA) [7,8]. This “collateral activity” was promptly employed to develop a sensitive, specific and rapid method for nucleic acid detection. CRISPR-Cas12a has been used to detect viruses and discriminate human papillomavirus (HPV) genotypes in either virus-infected human cell lines and clinical patient samples, and also for SNP genotyping [7,8]. At the same time, a CRISPR-Cas13-based nucleic acid detection system successfully detected Zika and Dengue viruses (ZIKV, DENV), bacterial isolates, antibiotic-resistant genes, human DNA genotypes, and cancer mutations [9]. However, the application of Cas12a for detecting both DNA and RNA pathogens was not hitherto reported.

**Figure 1.**
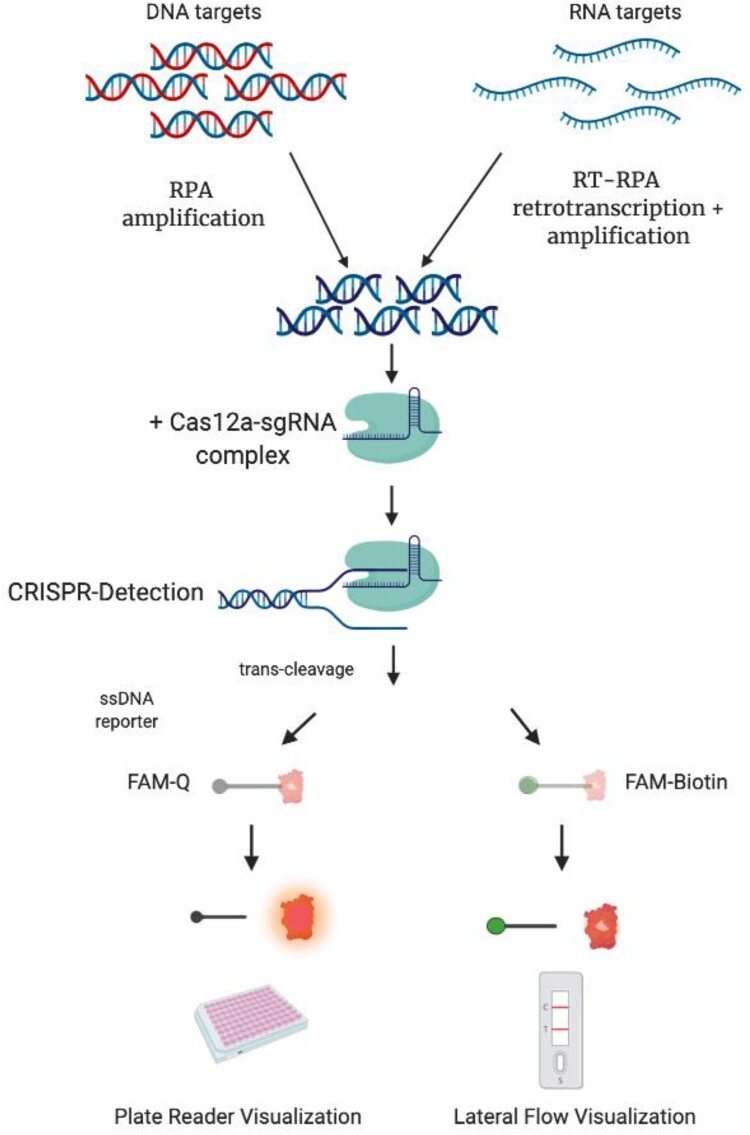
Schematic description of CRISPR-Cas detection protocol. To detect a DNA target sequence, a specific isothermal amplification step is first required. In the case of a RNA target, this step needs to be complemented with a reverse transcription reaction. A sgRNA sequence is specially designed, matching a region in the target DNA or cDNA. After the enzyme is loaded with the sgRNA, the amplicon is mixed with the Cas12a/sgRNA complex. A ternary complex only forms if the target DNA is present in the sample. Upon formation of the ternary complex, the quenched fluorescent ssDNA reporter is trans-cleaved, triggering a fluorescence signal that can be monitored with a plate reader. Alternatively, a FAM-Biotin labelled ssDNA reporter could be used, and results visualized with a lateral flow readout.

In this work, we show that the Cas12a endonuclease could be employed for detecting both DNA and, indirectly, RNA targets such as antibiotic-resistant genes, tropical Dengue (DENV) and Zika (ZIKV) viruses and endemic (HANTV) viruses, demonstrating the versatility of this platform regardless of the nature of nucleic acid (Figure 1).

## Materials and methods

### Design and synthesis of nucleic acid targets and sgRNA

DNA targets, from 200 up to 450 bp, were designed using a conserved zone of the sequence of the carbapenemase-encoding genes as a reference, available at the GenBank-NCBI database (KPC NC_014312.1; NDM, NC_023908.1; OXA-48, NC_019154.1). These targets were synthesized as gBlocks by Integrated DNA Technology (IDT) (San Diego, USA). gBlocks were amplified using NEBNext^®^ High-Fidelity 2X PCR Master Mix from New England Biolabs (NEB) (Massachusetts, USA) following the manufacturer's instructions. Additionally, RNA targets were designed using a 100 bp conserved zone of viral genomes (HANTV, NC_003466.1; ZIKV, MH900227.1; DENV, DQ863638.1) and synthesized as RNA by IDT (San Diego, USA). Synthetic RNA targets were reverse-transcribed with OneTaq^®^ RT–PCR Kit from NEB (Massachusetts, USA) using specific primers for cDNA conversion. Obtained cDNA was amplified using NEBNext^®^ High-Fidelity 2X PCR Master Mix (NEB). In all the cases, PCR products were visualized by staining after agarose gel electrophoresis, purified using QIAquick PCR Purification Kit from QIAGEN (Hilden, Germany), and quantified using NanoDrop^™^ Lite Spectrophotometer from Thermo Fisher (Massachusetts, USA).

All sgRNAs were designed using CRISPRscan Software from Giraldez’s Lab [10] based on LbCas12a (Cpf1) predicted guides. RNA constructs were synthesized by Synthego^TM^ (California, USA). Complete lists of gBlocks, ssRNAs, primers and sgRNAs are shown in Tables S1–S3 of the Supplemental Material.

### Recombinase polymerase amplification (RPA) reactions and primers design

Primers for RPA were designed using NCBI Primer-BLAST with the following restrictions: amplicon size (between 100 and 140 nt), primer melting temperatures (between 54°C and 67°C), and primer size (between 30 and 35 nt). Primers were then synthesized by IDT (Table S2). RPA reaction runs were carried out as instructed by TwistAmp^®^ Basic from TwistDx (Cambridge, United Kingdom) and RT-RPA was carried out adding 2.5 μl of M-MuLV Reverse Transcriptase (NEB) and 0.5 μl of murine RNase Inhibitor (NEB). Reactions were run with 1 μL of DNA or RNA templates from 10^−8^–10^−16^ M (or water for negative control) for 20–50 min at 37°C for RPA, and 42°C for RT-RPA, respectively. When indicated, 250 ng of total genomic DNA from MIA PaCa-2 cultured cells (ATCC^®^ CRL-1420^™^) was used as background.

### Target detection assays using a Fluorophore/Quencher (FQ)-labeled reporter

Lba Cas12a from *Lachnospiraceae bacterium* (NEB) was used in all CRISPR detections. Cas12a-sgRNA complexes were pre-assembled by incubating both components at a final concentration of 30 nM each, at room temperature for 10 min a solution containing 10X NEBuffer 2.1 (NEB) (100 mM NaCl 50 mM Tris-HCl,10 mM MgCl_2_, 1 mM DTT, pH 7.9) and 30 nM custom ssDNA FQ reporter substrates /56-FAM/TTATT/3IABKFQ (IDT) in a 40 μL final reaction volume. DNA/RNA Target detection assays were performed as above, but adding different target concentrations. For CRISPR detections 4 μL of purified DNA from 10^−8^–10^−16^ M was used as input. Reactions (40 μl, 384-well microplate format) were incubated in a fluorescence plate reader (SpectraMax M2 – Molecular Devices) for up to 90 min at 37°C with fluorescence measurements taken every 2 min (ssDNA FQ reporter λex: 485 nm; λem: 535 nm). For trans-cleavage rate determination, background-subtracted fluorescence values were calculated by subtracting fluorescence values obtained from reactions carried out in the absence of target.

### Clinical samples/ethical statements

Blood samples were collected from dengue infected patients (NS1 protein presence) in Posadas, Misiones, Argentina, as part of a study assessing arboviral distribution using molecular protocols. This project, and associated consent forms, have been evaluated and approved by the regional hospital ethical committee (CEIP: Provincial Ethical Committee in Research). RNA from serum samples of patients confirmed positive for DENV was extracted following the recommended methods used for RNA preparation previous to the qPCR assays, as described below. Samples were pre-tested using qPCR as described in Santiago et al.[11].

### Sample preparation

Whole blood was diluted 1:50 in PBS and RNase inactivation was performed by the addition of 100 and 1 mM of TCEP/EDTA, respectively to blood samples. This treatment was followed by a heat inactivation step at 95 °C for 10 min, as previously described [12]. When indicated, different concentrations of genomic ssRNA or DNA were included before heat inactivation to simulate real samples (spike samples), following standard protocols [13]. Nucleic acids were provided by Biobank (Bei Resources), as follows: genomic DNA (gDNA) from *Klebsiella pneumoniae* isolate (BEI-NR-15466), genomic RNA from Zika Virus (CDC-259359) and genomic RNA from Dengue Virus from ATCC (BEI-NR-32847).

### Reaction detection using a lateral low approach

A lateral flow system, using commercially available detection strips (Milenia Hybridetect 1, TwistDx, Cambridge, UK), was also employed as readout in order to detect the degradation of the previously described oligonucleotide probe. Briefly, after 1hr of CRISPR-Cas12a catalysis, reaction mixes were diluted 1:5 in Hybridetect Assay Buffer, and then strips were inserted and incubated for 5 min at room temperature. The strips were then removed and photographed using a smartphone camera.

### Statistics

Microsoft Excel 2016 was used for data analysis. GraphPad Prism v.8.1.2 (San Diego, USA) was used for graphic design and statistical tests. In all cases, error bars show Mean ± SEM (*n* = 3). When indicated, *t*-test and Bonferroni’s test were done.

## Results

### Limit of detection assessment using synthetic DNA targets

**Figure 2.**
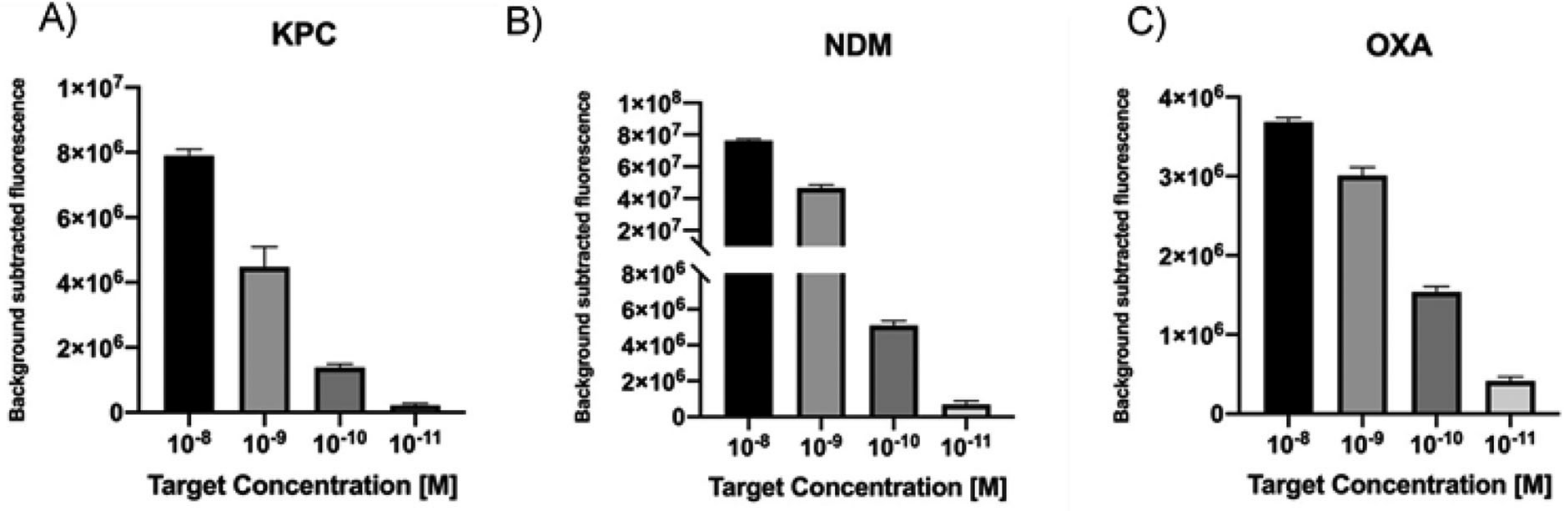
A–C. Detection limit assessment for synthetic DNA targets using the CRISPR-Cas12 platform. Fluorescence signal at 10 min with different target concentration is shown. Background subtracted fluorescence corresponds to sample minus control fluorescence, without target. Bars show mean ± SEM (*n *= 3).

In order to test the LbCas12a-based detection platform and corroborate the accuracy of sgRNAs design, we first used antimicrobial resistance (AMR) genes as DNA targets. KPC, NDM and OXA-48 like gene fragments were synthetized as dsDNA gBlocks. Furthermore, to determine the limit of detection (LoD) for this method, serial dilutions of the dsDNA targets in the range 10 nM to 10 pM were tested. Results show that when the concentration decreased, the fluorescence decreased proportionally (Figure 2(A–C)). Remarkably the system was able to detect at picomolar levels of each target in 10 min.

**Figure 3.**
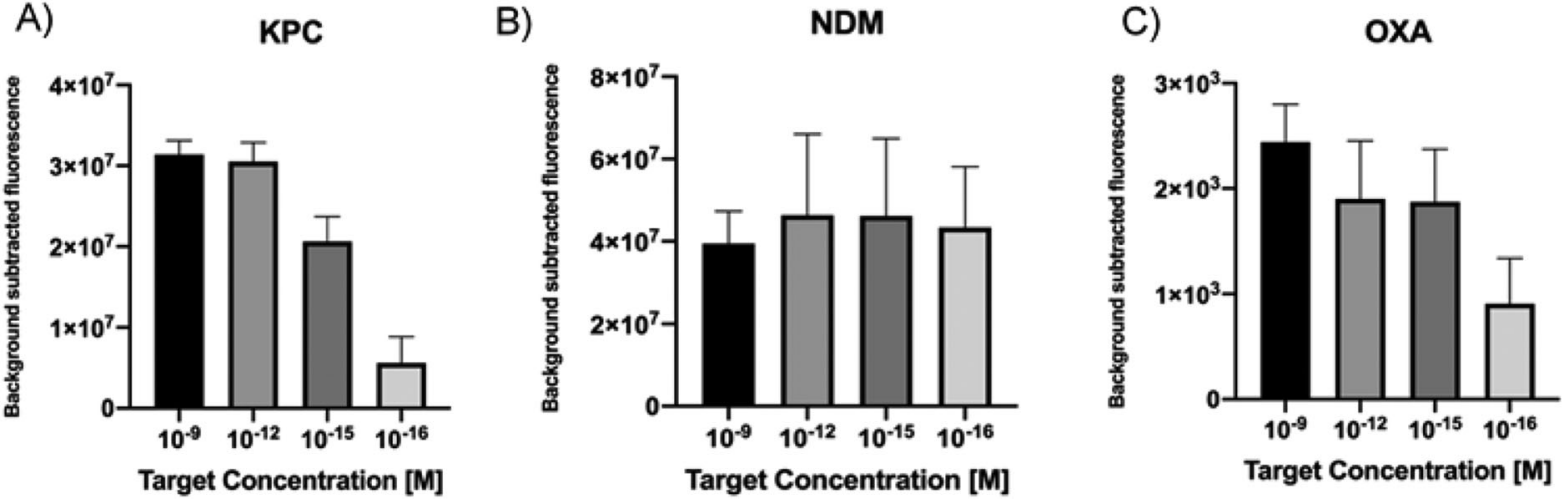
A–C. Detection limit assessment for synthetic DNA targets using a RPA-CRISPR-Cas12 combined strategy. Fluorescence values after 20 min of RPA and 30 min of CRISPR-Cas12 detection using as input different concentration of DNA targets. Background subtracted fluorescence corresponds to sample minus control fluorescence, without target. Bars show mean ± SEM (*n *= 3).

Taking into account that clinical samples represent a challenge for molecular diagnostics because of reaction inhibition due to the genomic DNA (gDNA) background and the low amounts of free target in the blood, some modifications were introduced in our protocol in order to test the performance of the CRISPR-LbCas12a platform. An isothermal pre-amplification step (RPA) was used initiate the nuclease reaction with higher target concentrations, using as template target serial dilutions in the nanomolar to attomolar range. Moreover, non-related bacterial gDNA was introduced in the sample and used as background control. Results showed that in all cases, target attomolar levels were detected in less than an hour (Figure 3(A–C)).

### Cross reaction test with unrelated DNA targets

**Figure 4.**
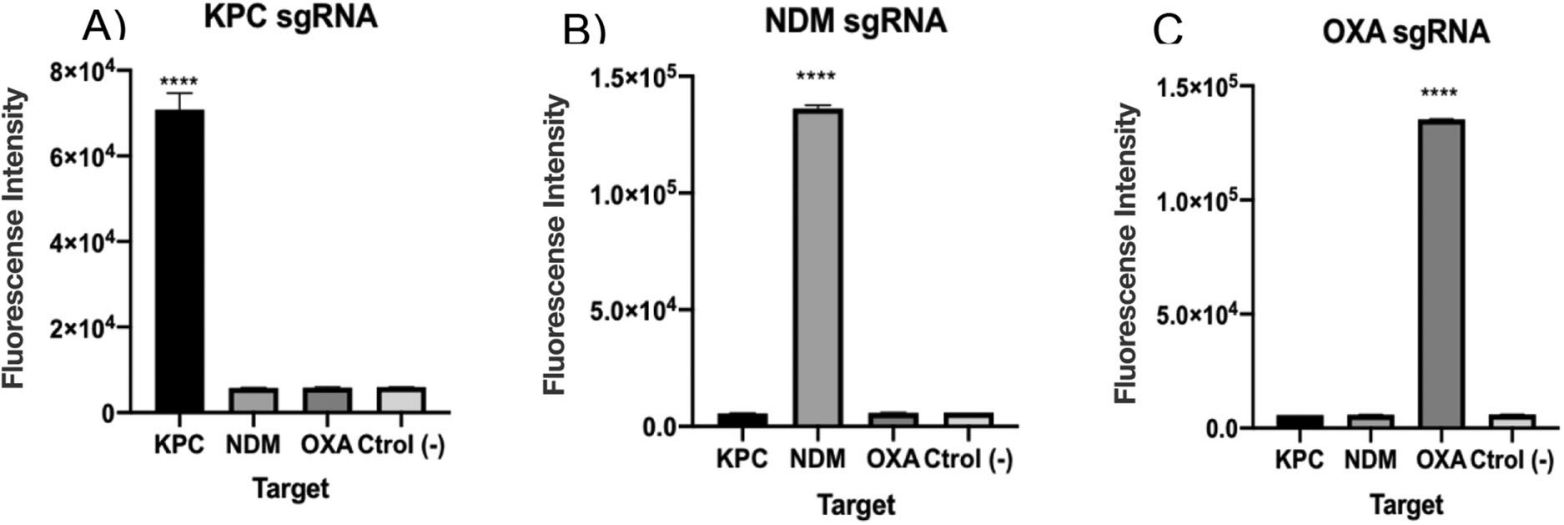
A–C. Cross reaction tests for each of the carbapenemase gene DNA targets. Fluorescence values after 15 min for 1 nM of synthetic DNA. The absence of a template was used as negative control. As seen, background fluorescence is in the range of negative samples. T-test (two tailed) was performed for each group against the control. Bars show mean ± SEM (*n *= 3). **** means *P *< 0.001 compared to control.

After confirming that each of the designed sgRNAs were efficient in detecting the selected DNA targets at clinical concentration levels, we performed specificity tests to demonstrate the absence of cross reaction with unrelated targets (either KPC, NDM and OXA as appropriate). To this end, LbCas12a enzyme was separately pre-incubated with each of the sgRNAs and then confronted to the cited DNA targets. Results showed that only the specific target matching the correct sgRNA was detected in each case (Figure 4(A–C)). In conclusion, LbCas12a thus catalysed ssDNA cleavage after site-specific dsDNA cutting guided by our designed sgRNA, and no cross-reaction was observed.

### Target detection using bacterial genomic DNA

**Figure 5.**
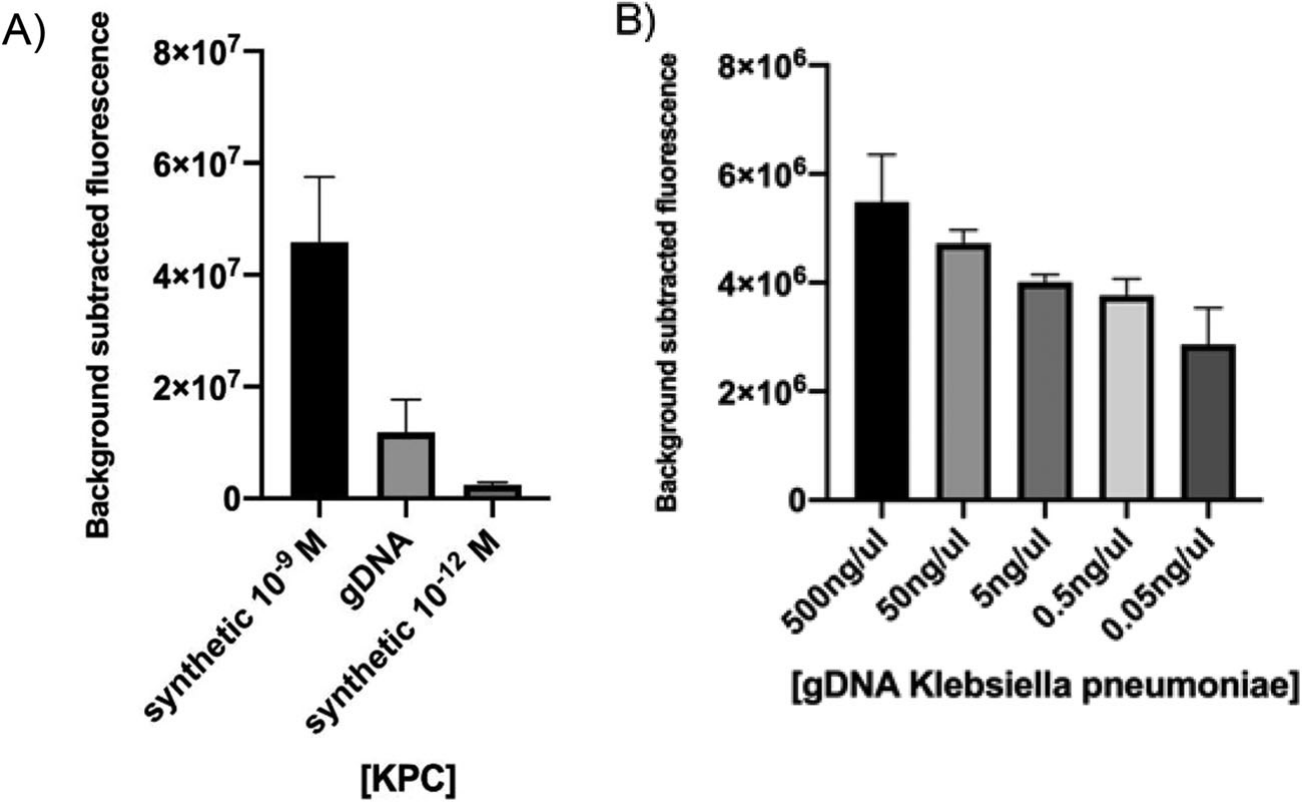
Detection of a sequence contained in bacterial gDNA. (A) CRISPR-Cas12 detection of the KPC gene encoded by a *Klebsiella pneumoniae* isolate was preceded by isothermal amplification. Reaction mixture using different concentrations of synthetic target was used for comparison. (B) CRISPR-Cas12 detection of the KPC gene in a spiked blood sample. Background subtracted fluorescence represent sample minus control fluorescence, without target. In both assays, bars represent fluorescence at 30 min and show mean ± SEM (*n *= 3).

To improve our detection method, it was necessary to go one step further towards the simulation of clinical samples. Hence, the next objective was checking if our platform had the capacity of detecting the KPC gene sequence belonging to a bacterial genome. To this end, *K. pneumoniae* BEI-NR-15466 gDNA was used as input for target isothermal amplification and CRISPR-based detection. Comparative results against synthetic DNA showed that the LbCas12a-sgRNA complex *in vitro* detected the selected target in 30 min (Figure 5(A)), thus confirming that the method could not only detect the corresponding sequence included in a fragment of 450 bp but also as part of the genome of a bacterial pathogen, which is above 5 Mb.

Then we decided to challenge the system to detect a KPC gene sequence of *K. pneumoniae* gDNA straight from a blood sample, simulating a patient with bacteraemia (spiked sample). For this assay, whole blood was chemically and thermally inactivated and gDNA was added prior to RPA. Results showed that our system was capable of detecting bacterial gDNA in 30 min directly from blood, without the need of previous purification steps (Figure 5(B)). Furthermore, fluorescence signal decreased proportionally with gDNA concentration.

### Limit of detection of synthetic RNA targets

Infections caused by viruses are very important to diagnose, especially in cases where they are asymptomatic, share the same symptoms (as DENV and ZIKV viruses) or in cases where the infections could be lethal as HANTAV [14,15]. Given their clinical importance, we designed sgRNAs and performed tests to detect RNA targets located within the sequences of each of the afore mentioned viruses.

**Figure 6.**
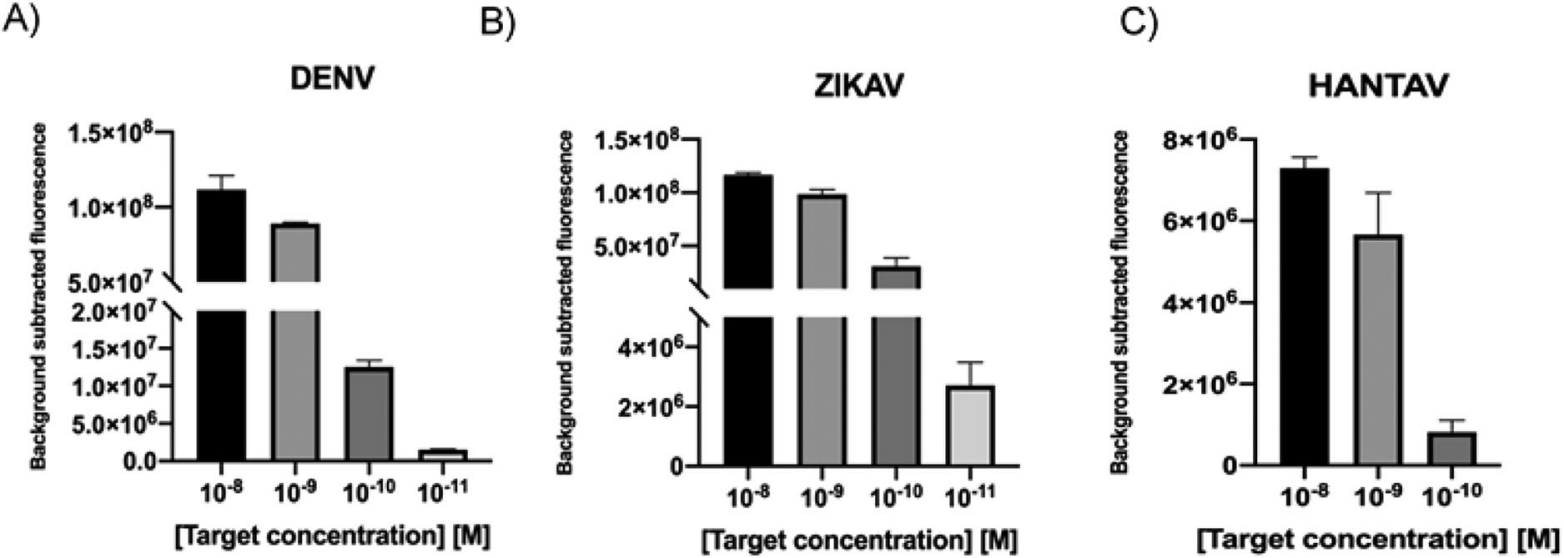
A–C. Detection limit assessment for synthetic RNA targets using the CRISPR-Cas12 platform. Fluorescence signal at 20 min with different target concentration of synthetic ssRNA reverse transcribed into cDNA is shown. Background subtracted fluorescence corresponds to sample minus control fluorescence, without target. Bars show mean ± SEM (*n *= 3). * only the results obtained with the more efficient sgRNA are shown.

Following a similar approach as for DNA targets, LoD determination was carried out in first place. Viral ssRNA was reverse transcribed into cDNA and used as input in LbCas12a-containing reaction mixtures. Serial dilutions in the range 10 nM to 10 pM were tested. Results showed that the CRISPR-Cas-based system was able to detect targets up to a picomolar concentration range in 30 min, reaching similar levels as those found for dsDNA targets (Figure 6(A–C)).

**Figure 7.**
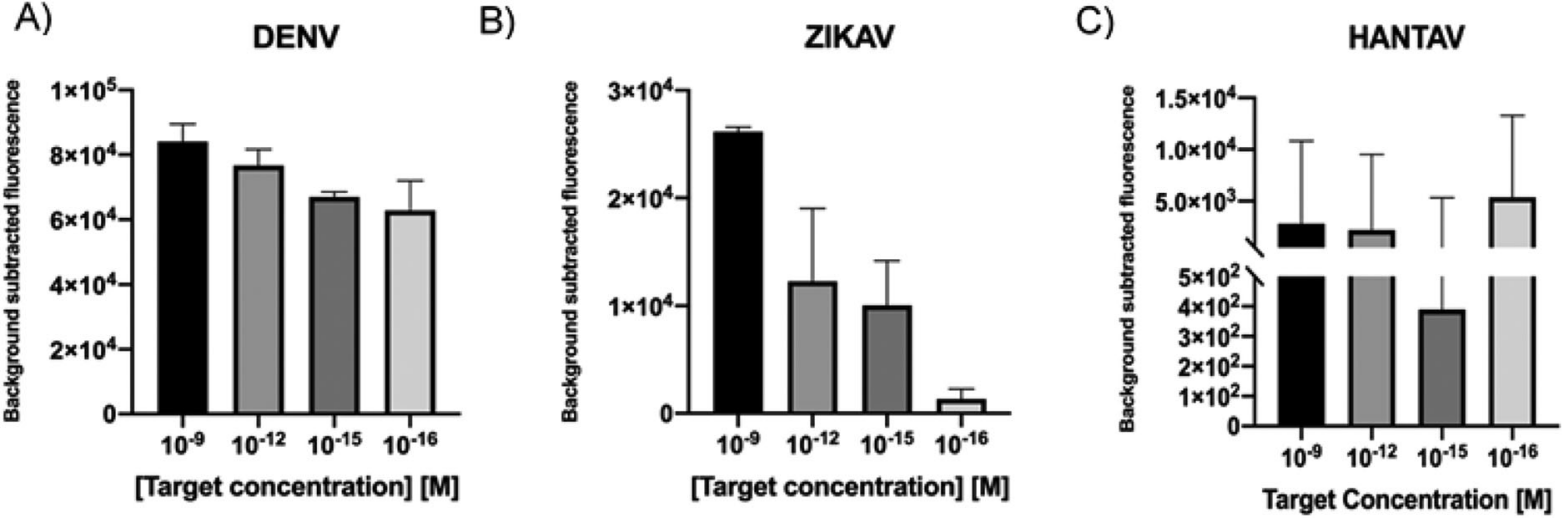
A–C. Detection limit assessment for synthetic RNA targets using a RT-RPA-CRISPR-Cas12 combined strategy. Fluorescence values after 30 min of RT-RPA and 30 min of CRISPR-Cas12 detection for serial dilutions of RNA targets derived from synthetic templates. Background subtracted fluorescence corresponds to sample minus control fluorescence, without target. Bars shows mean ± SEM (*n *= 3).

Considering the heterogeneity of viral load in clinical samples [16], with some of them having titres below the detection limit of the CRISPR-Cas system, a RPA step was included in our workflow to ensure target detection. In this case, ssRNA was reverse transcribed at the same time amplification occurs, thanks to the addition of MuLV enzyme to the RPA mix (RT-RPA). 200 ng of random RNA molecules used as background was added into the mixture, trying to simulate the characteristic noise in a sample. Results showed that in all cases, the CRISPR-Cas based-detection platform could detect RNA targets at the attomolar level (Figure 7(A–C)).

### Cross reaction test with unrelated RNA targets

**Figure 8.**
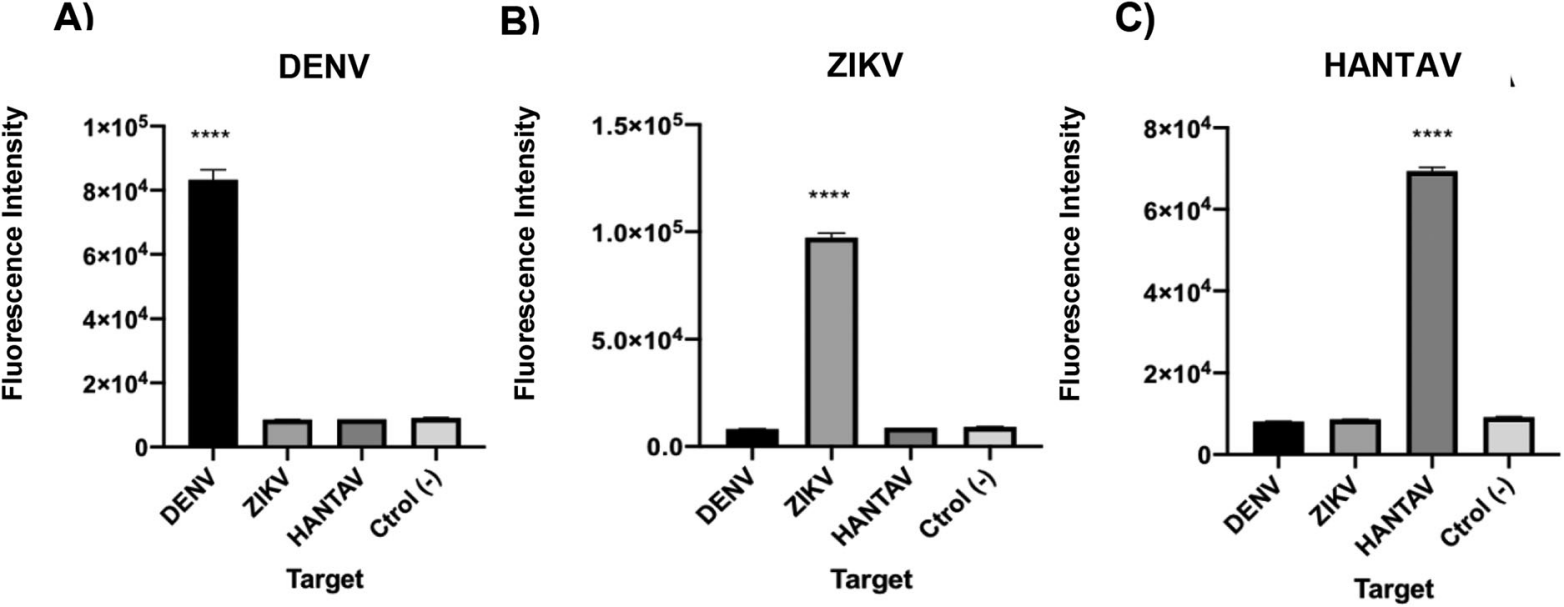
A–C. Cross reaction tests for each of the viral RNA targets. Fluorescence values after 30 min of detection for 1 nM of synthetic ssRNA reverse transcribed into cDNA. The absence of a template was used as negative control. T-test (two tailed) was performed for each group against the control. Bars show mean ± SEM (*n *= 3). *** means *P *< 0.001 compared to control.

After demonstration that the CRISPR-Cas based system was able to detect synthetic RNA targets, after a reverse transciption step, it was important to demonstrate that it did not show cross reaction with other targets given the implications at the clinical level, since infections caused by these viruses present similar symptoms. Results showed that in each case, assays only detected the specific sequence and no cross-reaction was observed (Figure 8(A–C)). In conclusion, each LbCas12a-sgRNA complex was able to specifically detect synthetic nucleic acid targets independently of their nature, avoiding off-targets.

### Detection of viral genomic RNA

**Figure 9.**
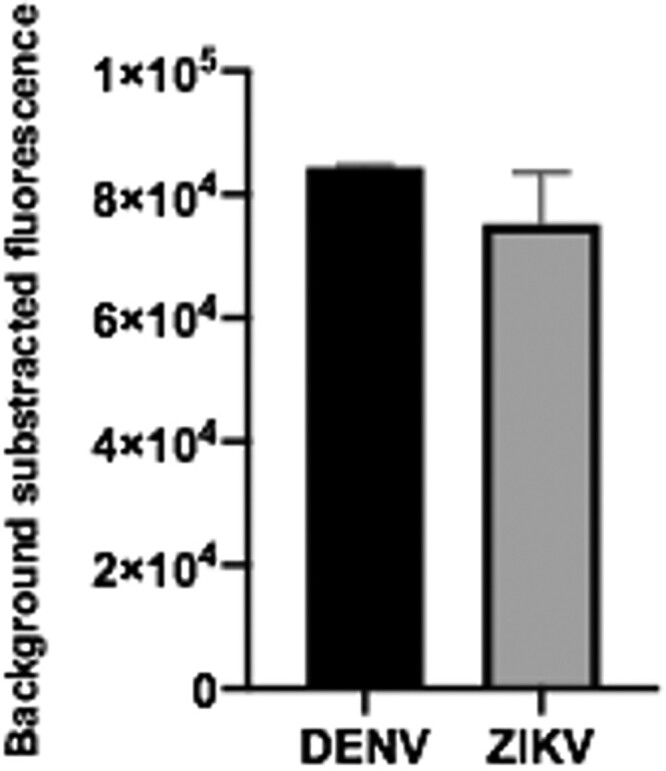
Viral gRNA target detection. Genomic ssRNA from ZIKV and DENV were used as input. RT-RPA amplification plus CRISPR-Cas12 detection was then done. Data correspond to 30 min reaction time. Bars show mean ± SEM (*n *= 2).

We then decided to test if the sgRNAs designed for the detection of synthetic RNAs corresponding to ZIKV and DENV, were also efficient when the whole system was faced with viral genomic RNA. Genomic ssRNA was thus used as input in RT-RPA amplification followed by CRISPR-Cas-mediated detection. Results showed that the expected genomic target sequences could be detected for both viruses (Figure 9). These assays demonstrate that direct RT-RPA can be performed to obtain amplified cDNA using as template the ssRNA, and that the system can detect not only synthetic RNA sequences but also the actual sequence contained in the viral genome.

Viral subtypes determination is an important matter, since symptoms and lethality may variate when co-infection or re-infection occur. A typical case for which this information results critical is that of DENV, which presents 4 different subtypes. In view of this situation, we decided to configure the CRISPR-Cas system with a sgRNA (sgRNA_DENV1/2/3) previously designed to detect subtypes 1, 2 and 3 [12]. DENV genomic RNA was used as input in a RT-RPA reaction and then followed by CRISPR-Cas detection. A cross-reaction test with ZIKV genomic RNA was also included. Results showed that the LbCas12a-sgRNA complex could effectively detect viral subtypes 1, 2 and 3 but, as expected, -not subtype 4 (Supplementary Figure S1). In sum, we showed the capacity of this system to potentially distinguish between DENV subtypes, demonstrating no cross-reaction with ZIKV.

### Detection of DENV in patient samples coupled with a lateral flow visualization system

**Figure 10.**
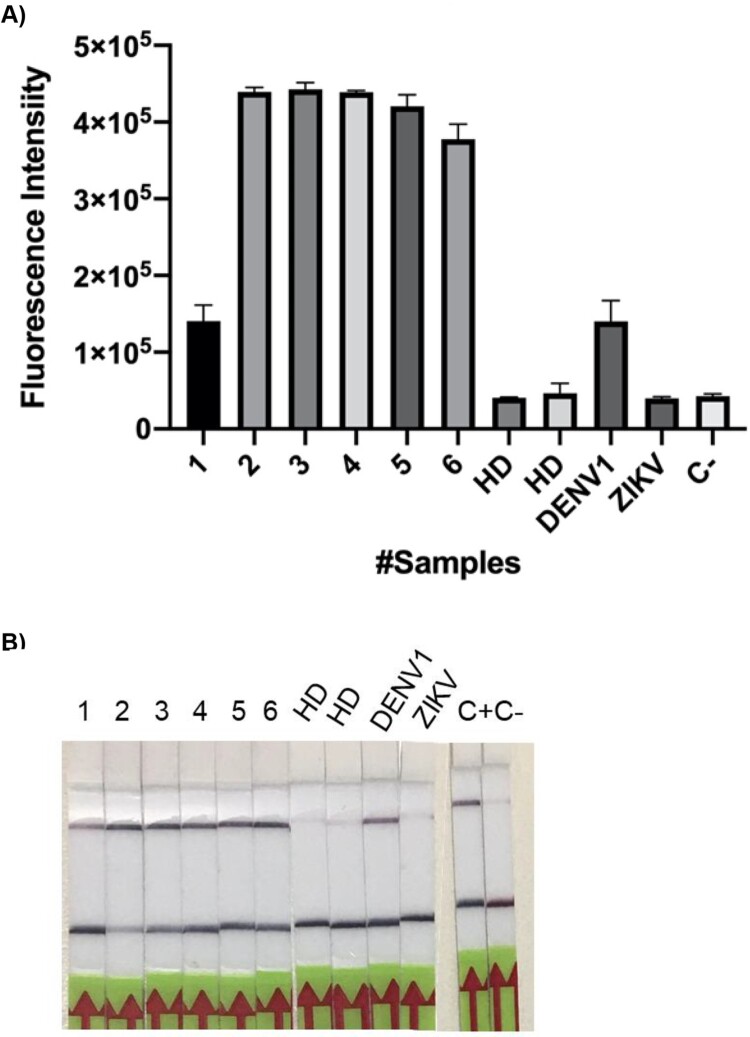
A–B. DENV detection in patient samples visualized by both fluorescence and lateral flow readout systems. (A) RT-RPA amplification followed by CRISPR-Cas12 detection was done on clinical samples: 1–6, positive for DENV according to qPCR assays; 7–8 obtained from healthy donors (HD). Fluorescence signal was measured after 140 min of reaction. DENV ssRNA from CDC was used as positive control. Cross reaction tests with ZIKV gRNA from was also performed. Reaction mixture without RNA input addition was used as negative control (C-). Bars show mean ± SEM (*n *= 2). (B) The lateral Flow assay was carried out, in parallel with the fluorescent measure, and pictures were taken after 5 min.

In order to validate our design, we next decided to test the system with clinical samples. The CRISPR/Cas-based platform was then compared, face to face against RT-qPCR, the gold standard technique for DENV detection (Figure 10). Of a total of 8 clinical samples tested by RT-qPCR, 6 had been previously confirmed positive (#1-6), and 2 (# 7 and 8) corresponded to healthy donors without antecedents of DENV infection, and therefore expectedly negative. In addition, positive CDC controls for DENV (#9) and a cross-reaction control with Zika virus (#10) were added. As observed in Figure 10(A), the test showed a sensitivity, specificity and positive prediction value of 100% (6/6). It is worth noting that with the exception of sample 1, the remaining samples were positive at 25 min of reaction. Regarding negative controls, CRISPR detection matches with the results of RT-qPCR. No cross-reaction was observed with ZIKV control.

When the readout system was changed to test strips, the results were robust and sensitive. Figure 10(B) shows a 100% correlation between the results of the fluorescence test and those obtained with the strips.

## Discussion

Cas12 is a DNA-targeting enzyme, while Cas13 recognizes RNA targets. However, regardless of their nature, targets can be interconverted during nucleic acid amplification (e.g. DNA to RNA by transcription or RNA to DNA by reverse-transcription), enabling these systems to detect both DNA and RNA targets. As far as we know, no other works have reported the use of Cas12a to detect DNA or RNA targets the way we describe here.

The entire process of CRISPR-Cas-detection systems includes sample pre-treatment, nucleic acid amplification, CRISPR/Cas-mediated binding/cleavage, and signal detection. The total time required for each method varies from 2–5 h for SHERLOCK and about 2 h for DETECTR [7,15,20]. Here we show that in less than 2 h, detection could be done reaching attomolar levels. Regarding sample treatment, incubation times depend on the types sample and could be as short as 10 min, as demonstrated for blood samples with the HOLMES test [8]. The speed of nucleic acid amplification strongly correlates with the selected method, where RPA methods need only 15–60 min. The Cas-mediated reaction is the key step in CRISPR/Cas-based detection, and the assay time is 1 h for DETECTR, and 30 min to 3 h for SHERLOCK, mostly depending on the selected Cas effectors [7,15,20]. Here, we showed that shorter incubation times can be used for signal detection. If a plate reader is used, measurements are obtained every 2 min and the trend is already manifested within 10 min.

In this work, we were able to detect synthetic targets, in some cases up to picomolar levels without a previous step of isothermal amplification. When we incorporated isothermal amplification into our workflow, the LoD was lowered to attomolar levels in all targets. Other authors reach the same sensitivity for different targets [7] or using different endonucleases [17]. Interestingly, since this method attains attomolar levels of target detection, we reach clinically relevant detection levels [18,19].

In terms of antimicrobial resistance, with the increase of carbapenemase-producing enterobacteriaceae (CPE), the treatment of severe bacterial infections has become more difficult, especially in regions of high prevalence. Among the different carbapenemases, OXA β-Lactamases (OXA), *K. pneumoniae* carbapenemase (KPC), and New Delhi metallo-beta-lactamase (NDM) are the most important worldwide [20]. Using the CRISPR-Cas12a system we were able to detect KPC, NDM and OXA genes when present as synthetic targets. Additionally, we tested the cross reaction between these targets. The system is very specific, detecting only the target matching the sgRNA. This is of great importance since carbapenem resistance could be conferred by different genes, which would slow down the medical diagnostics process. Also, we demonstrated that the CRISPR-Cas12a platform could detect the KPC gene encoded by a *K. pneumoniae* isolate even in complex samples such as blood, emulating a clinical sample. In this line, the potential of the Cas12a effector to detect targets in patient samples has been recently demonstrated by other authors [21].

All published CRISPR-based detection methods intended for bacterial pathogens require pre-treatment of samples to facilitate subsequent amplification and detection. Among these pre-treatments, heating could be the simplest and cheapest procedure; this was used in the HUDSON method prior to the SHERLOCK detection approach, and has demonstrated effectiveness on serum, saliva, and urine samples [22]. A similar procedure for direct detection in saliva or cell samples before PCR amplification, has been applied for HOLMES [12]. In our system, the combination of chemical and heat treatments exhibited similar results.

Among different ssRNA viruses, we tested DENV, ZIKV and HANTAV, the latter constituting a novel target for CRISPR/Cas-dependent detection methods. Other methods have been designed for the detection of DENV and ZIKV, such as SHERLOCK. However, this method uses an additional step, reconverting the amplified cDNA into RNA post reverse transcription [9,12,17]. This procedure involves two biochemical reactions, while our Cas12a-based platform only requires one (just converting RNA into cDNA). Therefore, our method is timesaving, especially in the case of RNA targets. Additionally, we demonstrated that the CRISPR-Cas12a platform is able to detect ZIKV genomic ssRNA and has the capability of distinguishing between DENV subtypes, which results of high relevance for the patient outcome [23]. Noteworthy, when the assays were repeated using patient samples, results showed comparable accuracy with those obtained by qRT-PCR, the molecular gold standard. Lastly, our method was adapted to a different reporter system, similar to that employed for pregnancy tests, for which results could be read on commercial lateral flow strips. This type of readout permits an affordable, rapid and precise nucleic acid detection method that can be used almost anywhere. This is particularly important in middle or low-income countries, where expensive equipment for signal detection is not available.

## Conclusions

Several nucleic acid detection methods have been rapidly developed with various Cas effectors, which could be considered as the tip of the iceberg for this novel biosensing technology in the diagnostics field. Most of these CRISPR/Cas-detecting systems provide several advantages such as simplicity to be developed/re-developed, ultra-high resolution which enables to distinguish single-base variations, and a detection limit attaining at least fM but mostly aM target concentrations. Specifically, our results showed that the Cas12a-effector could be used both with DNA or RNA targets, reducing the steps of the detection process for RNA targets compared with Cas13. In addition, we demonstrated that the CRISPR-Cas12 based detection method can be used in a variety of targets in a paper strip without the requirement of sophisticated instrumentation. All in all, we expect that the continuous development of CRISPR/Cas-detection will allow molecular diagnostics to be broadly applied in the near future.

## Supplementary Material

Supplemental Material
