# Supplementary material for: CRISPR-based platform for carbapenemases and emerging viruses detection using Cas12a (Cpf1) effector nuclease
Source: Emerg Microbes Infect. 2020 Jun 2;9(1):1140–8. doi: 10.1080/22221751.2020.1763857 (PMC7448918; doi:10.1080/22221751.2020.1763857)
Supplement: Supplemental Material [file TEMI_A_1763857_SM5371.doc]

**Table S1. List of synthetic DNA and RNA targets used in this study.**

| Name | Sequence |
| --- | --- |
| KPC gBlock | TTCAAGGGCTTTCTTGCTGCCGCTGTGCTGGCTCGCAGCCAGCAGCAGGCCGGCTTGCTGGACACACCCATCCGTTACGGCAAAAATGCGCTGGTTCCGTGGTCACCCATCTCGGAAAAATATCTGACAACAGGCATGACGGTGGCGGAGCTGTCCGCGGCCGCCGTGCAATACAGTGATAACGCCGCCGCCAATTTGTTGCTGAAGGAGTTGGGCGGCCCGGCCGGGCTGACGGCCTTCATGCGCTCTATCGGCGATACCACGTTCCGTCTGGACCGCTGGGAGCTGGAGCTGAACTCCGCCATCCCAGGCGATGCGCGCGATACCTCATCGCCGCGCGCCGTGACGGAAAGCTTACAAAAACTGACACTGGGCTCTGCACTGGCTGCGCCGCAGCGGCAGCAGTTTGTTGATTGGCTAAAGGGAAACACGACCGGCAACCACCGCATCCGCGCGGCGGTGCCGGCAGACTGGGCAGTCGGAGACA |
| NDM gBlock | CCAAATTAAGATCATCTATTTACTAGGCCTCGCATTTGCGGGGTTTTTAATGCTGAATAAAAGGAAAACTTGATGGAATTGCCCAATATTATGCACCCGGTCGCGAAGCTGAGCACCGCATTAGCCGCTGCATTGATGCTGAGCGGGTGCATGCCCGGTGAAATCCGCCCGACGATTGGCCAGCAAATGGAAACTGGCGACCAACGGTTTGGCGATCTGGTTTTCCGCCAGCTCGCACCGAATGTCTGGCAGCACACTTCCTATCTCGACATGCCGGGTTTCGGGGCAGTCGCTTCCAACGGTTTGATCGTCAGGGATGGCGGCCGCGTGCTGGTGGTCGATACCGCCTGGACCGATGACCAGACCGCCCAGATCCTCAACTGGATCAAGCAGGAGATCAACCTGCCGGTCGCGCTGGCGGTGGTGACTCACGCGCATCAGGACAAGATGGGCGGTATGGACGCGCTGCATGCGGCGGGG |
| OXA-48 gBlock | CGAAGCCAATGGTGACTATATTATTCGGGCTAAAACTGGATACTCGACTAGAATCGAACCTAAGATTGGCTGGTGGGTCGGTTGGGTTGAACTTGATGATAATGTGTGGTTTTTTGCGATGAATA |
| DENV ssRNA target | UGACGAAGACCAUGCUCACUGGACAGAAGCAAAAAUGCUGCUGGACAACAUCAACACACCAGAAGGGAUUAUACCAGCUCUCUUUGAACCAGAAAGGGAG |
| ZIK ssRNA target | CCACACUGGAACAACAAAGAAGCACUGGUAGAGUUCAAGGACGCACAUGCCAAAAGGCAAACUGUCGUGGUUCUAGGGAGUCAAGAAGGAGCAGUUCACA |
| HANT ssRNA target | AGAGGCAACUUGCAGAUUUGGUGGCAGCUCAAAAAUUGGCUACAAAACCAGUUGAUCCAACAGGGCUUGAGCCUGAUGAUCAUCUAAAGGAAAAAUCAUC |

**Table S2. List of sgRNA sequences used in this study.**

| Name | Sequence |
| --- | --- |
| sgRNA_KPC | UAAUUUCUACUAAGUGUAGAUUUGCUGAAGGAGUUGGGCGGCCC |
| sgRNA_NDM | UAAUUUCUACUAAGUGUAGAUGCGAUCUGGUUUCCGCCAGCUA |
| sgRNA_OXA | UAAUUUCUACUAAGUGUAGAUAGCCCGAAUAAUAUAGUCRCCAU |
| sgRNA_DENV1/2/3 | UAAUUUCUACUAAGUGUAGAUAUUAGAGAGCAGAUCUCUGAUGA |
| sgRNA_DENV4 | UAAUUUCUACUAAGUGUAGAUAAUAGAGAGCAGAUCUCUGGAAA |
| sgRNA_ZIKV | UAAUUUCUACUAAGUGUAGAUCCUUUUGGCAUGUGCGUCCUUGA |
| sgRNA_HANTV | UAAUUUCUACUAAGUGUAGAUGAUGAUCAUCAGGCUCAAGCCCU |

**Table S3. Complete list of Primers used in this study.**

**PCR**

| Name | Forward | Reverse |
| --- | --- | --- |
| KPC_PCR | AAAAATGCGCTGGTTCCGTG | CCAGACGGAACGTGGTATCG |
| NDM_PCR | TTGCGGGGTTTTTAATGCTGAA | GTCCATACCGCCCATCTTGTC |
| OXA_PCR 1a | GAAGCCAATGGCGACTATATTATTCGGGC | CCACACATTATCATCAAGTTCAACC |
| OXA_PCR 1b | GAAGCCAATGGTGACTATATTATTCGGGC | CCACACATTATCATCCAGTTCAACC |

**RT-PCR**

| Name | Forward | Reverse |
| --- | --- | --- |
| DENV_PCR | TGACGAAGACCATGCTCACT | CTCCCTTTCTGGTTCAAAGAG |
| ZIKV_ PCR | CCACACTGGAACAACAAAGAAGC | TGTGAACTGCTCCTTCTTGACT |
| HANTVPCR | GAGGCAACTTGCAGATTTGGT | CTCAGAGATGATTTTTCCTTTAGAT |

**RPA**

| Name | | Forward | Reverse |
| --- | --- | --- | --- |
| KPC_RPA | AGCTGTCCGCGGCCGCCGTGCAATA | | TCAGCCCGGCCGGGCCGCCCAACTCCTTC |
| NDM_RPA | | CTGAGCGCATTAGCCGCTGCATTGATG | TGCCCCGAAACCCGGCATGTCGAGATAGGA |
